# Prediction of allograft function in pre-transplant kidneys using sound touch elastography (STE): an ex vivo study

**DOI:** 10.1186/s13244-024-01837-y

**Published:** 2024-10-11

**Authors:** Fu-shun Pan, Dao-peng Yang, Guo-dong Zhao, Shu-qi Huang, Yan Wang, Ming Xu, Jiang Qiu, Yan-ling Zheng, Xiao-yan Xie, Gang Huang

**Affiliations:** 1 Department of Medical Ultrasonics, Institute of Diagnostic and Interventional Ultrasound, The First Affiliated Hospital of Sun Yat-Sen University, Guangzhou, China; 2 Organ Transplant Center, The First Affiliated Hospital of Sun Yat-Sen University, Guangzhou, China; 3 Guangdong Provincial Key Laboratory of Organ Donation and Transplant Immunology, Guangzhou, China; 4 Guangdong Provincial International Cooperation Base of Science and Technology (Organ Transplantation), Guangzhou, China; 5 Department of Pathology, The First Affiliated Hospital of Sun Yat-sen University, Guangzhou, China

**Keywords:** Donor Kidney, Sound touch elastography, Allograft function, Remuzzi score

## Abstract

**Background:**

The purpose of the study was to evaluate renal quality and predict posttransplant graft function using ex vivo sound touch elastography (STE).

**Methods:**

In this prospective study, 106 donor kidneys underwent ex vivo STE examination and biopsy from March 2022 to August 2023. The mean stiffness of the superficial cortex (STE_sc_), deep cortex (STE_dc_), and medulla (STE_me_) was obtained and synthesized into one index (STE) through the factor analysis method. Additionally, 100 recipients were followed up for 6 months. A random forest algorithm was employed to explore significant predictive factors associated with the Remuzzi score and allograft function. The performance of parameters was evaluated by using the area under the receiver operating characteristic curve (AUC).

**Results:**

STE had AUC values of 0.803 for diagnosing low Remuzzi and 0.943 for diagnosing high Remuzzi. Meanwhile, STE had an AUC of 0.723 for diagnosing moderate to severe ATI. Random forest algorithm identified STE and Remuzzi score as significant predictors for 6-month renal function. The AUC for STE in predicting postoperative allograft function was 0.717, which was comparable with that of the Remuzzi score (AUC = 0.756). Nevertheless, the specificity of STE was significantly higher than that of Remuzzi (0.913 vs 0.652, *p* < 0.001). Given these promising results, donor kidneys can be transplanted directly without the need for biopsy when STE ≤ 11.741.

**Conclusions:**

The assessment of kidney quality using ex vivo STE demonstrated significant predictive value for the Remuzzi score and allograft function, which could help avoid unnecessary biopsy.

**Critical relevance statement:**

Pre-transplant kidney quality measured with ex vivo STE can be used to assess donor kidney quality and avoid unnecessary biopsy.

**Key Points:**

- STE has significant value for diagnosing low Remuzzi and high Remuzzi scores.
- STE achieved good performance in predicting posttransplant allograft function.
- Assessment of kidney quality using ex vivo STE could avoid unnecessary biopsies.

**Graphical Abstract:**

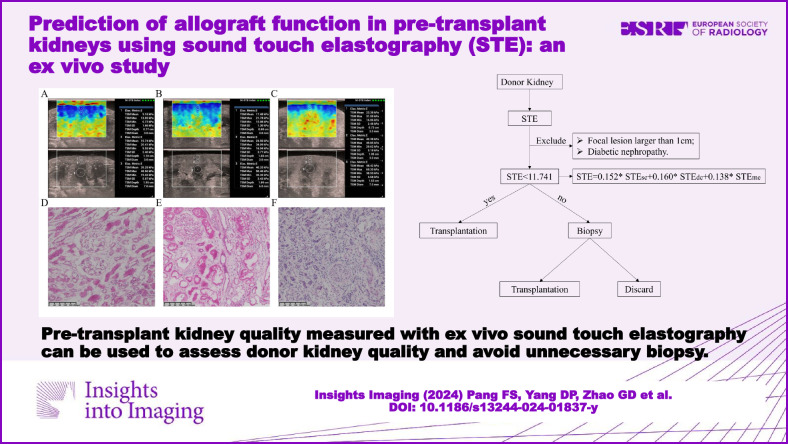

## Background

Renal transplantation remains the most cost-effective and preferable treatment for chronic kidney disease [1], but is still limited by donor shortage. One possibility for extending the pool of available kidneys is to consider expanded criteria donors (ECD) [2], such as elderly donors. However, transplantation of ECD kidneys is associated with an increased risk of delayed graft function (DGF), longer length of stay, and worse allograft function [3]. Therefore, utilization of marginal kidneys requires nuanced graft evaluation.

Currently, pre-transplant kidney quality assessment predominantly relies on renal biopsy and donor characteristics, such as the kidney donor profile index (KDPI). Remuzzi score for pre-implantation biopsy is considered the main standard for kidney graft evaluation [4]. However, as an invasive procedure, renal biopsy is associated with a series of complications, such as hematomas, arteriovenous fistula, and transplant loss in extreme cases [5]. Besides, histological examination is subject to sampling variability as it provides highly localized information. KDPI served as a clinical scoring system by providing an estimate of the posttransplant outcome [6]. But KDPI is constrained by its poor predictive accuracy and superior predictive models should be created [7]. Therefore, the development of a non-invasive and reproducible method for predicting Remuzzi score and allograft function is imperative.

Medical imaging has a significant role in the evaluation of functional and morphological information about the kidney [8]. Recent advancements in magnetic resonance imaging (MRI) and elastography (MRE) have shown promising potential in the noninvasive evaluation of post-transplant renal fibrosis [9]. However, limited accessibility and expense may hinder its widespread use. Additionally, stiffness measurements obtained by MRI and MRE can be affected by renal hemodynamic and structural factors [10]. US is the most useful imaging modality for evaluating the condition of the kidney. Over the past decade, the transabdominal application of various noninvasive ultrasound elastography techniques, has emerged as a promising method to quantify post-transplant fibrosis [11–13]. Nevertheless, the utility of elastography in post-transplant renal fibrosis assessment remains controversial due to various interfering factors, such as skin allograft distance [14] and renal perfusion [15].

On the contrary, kidneys are freed from the aforementioned interfering factors in the ex vivo setting, presenting an ideal scenario for elastography imaging. To the best of our knowledge, there have been no reports on the utilization of elastography for the assessment of pre-transplant kidney quality in an ex vivo setting. Thus, we hypothesize that ex vivo STE can provide real-time and objective information about the kidney, which can improve the accuracy of pre-transplant kidney quality evaluation.

As the latest elastography technique, sound touch elastography (STE) employs ultra-wideband technology to generate shear waves, allowing for the scanning of the entire kidney. STE has demonstrated high stability and reliability in the evaluation of the thyroid and liver [16, 17]. Therefore, the purpose of this study was to investigate the predictive value of ex vivo renal STE measurements for post-transplant renal function.

## Materials and methods

### Study design

This prospective study, which enrolled 129 donor kidneys that had undergone a preimplantation biopsy, was approved by our institutional ethics committee ([2023]205). The exclusion criteria for this study included: (1) specimen contained less than ten glomeruli and two small blood vessels; (2) diabetic nephropathy; (3) the presence of lesions larger than 1 cm in the central part of the kidney; (4) loss to follow-up; and (5) acute rejection occurring within six months postoperative.

### US and STE procedures

The donor's kidneys were transported and preserved using the traditional static cold technique. Following surgical reconditioning, the kidneys were immersed in a tank containing a saline solution mixed with ice. The STE procedures were performed by two experienced sonographers (F.-s.P. and D.-p.Y.) using a Resona 7 ultrasound system (Mindray, Shenzhen, China) equipped with a L14-5U linear array transducer (5–14 MHz). The operators were blinded to the clinical information. First, a US examination was performed to evaluate the allograft morphologic characteristics. When capturing the echogenicity of the renal cortex by histogram software (Mindray, Shenzhen, China), the B-mode ultrasound settings were standardized, with a dynamic range set to 135, gain adjusted to 80%, and the time gain compensation curve positioned at the center (Supplementary Fig. 1).

**Fig. 1:**
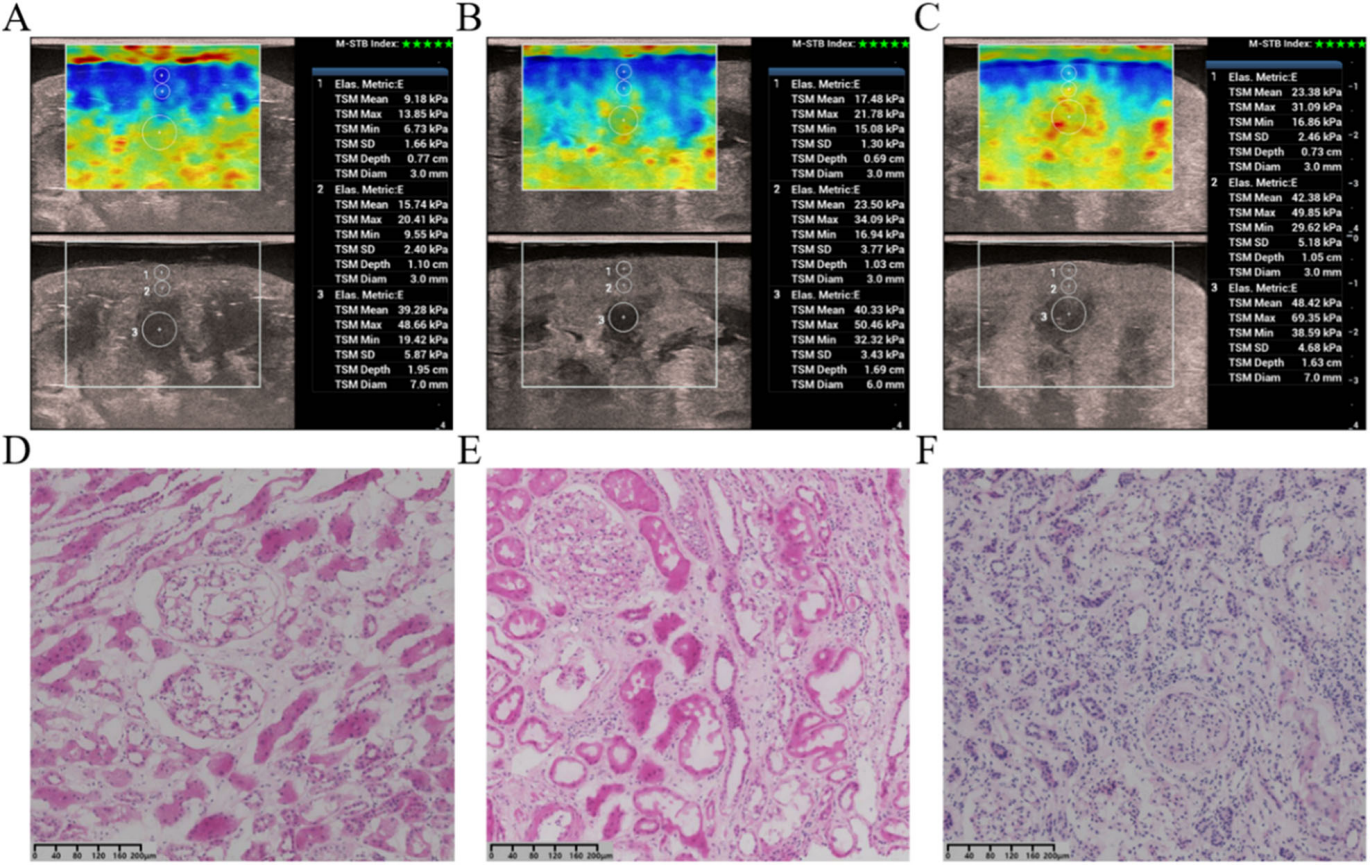
STE and histologic images for low, moderate, and high Remuzzi scores. **A**, **D** STE_sc_ = 9.18 kPa, STE_dc_ = 15.7 kPa, STE_me_ = 39.28 kPa, Remuzzi score = 0, GS = 0, IF = 0, TA = 0, and AS = 0. **B**, **E** STE_sc_ = 17.48 kPa, STE_dc_ = 23.50 kPa, STE_me_ = 40.33 kPa, Remuzzi score = 1, GS = 1, IF = 1, TA = 1, and AS = 1. **C**, **F** STE_sc_ = 23.38 kPa, STE_dc_ = 42.38 kPa, STE_me_ = 48.42 kPa, Remuzzi score = 9, GS = 3, IF = 2, TA = 2, and AS = 2

Next, an STE examination was performed (Fig. 1A–C). Operators gently hold the donor kidney in the hand and keep it from tilting, without applying additional force. To avoid any mechanical compression artifact, the probe was delicately positioned approximately 2 mm above the midpoint of the kidney. The color box was positioned within the cortex and medulla, oriented perpendicularly to the renal capsule to minimize the anisotropy effect. The STE settings were standardized with an elasticity range set to 0–75 kPa and the color box was sized at 4 cm in width and 3 cm in height. Following a brief period of immobilization, the image was frozen and stored. At least five measurements were taken for each kidney, and the entire STE examination lasted between 10 min and 15 min. All measurements were recorded and used for subsequent analyses.

### Image analysis

We determined the size of the ROI of STE based on the thickness of the cortex and medulla. A region of interest (ROI) was delineated for the superficial cortex, another ROI for the deep cortex, and a separate ROI for the medulla. At each phase, ROIs with a standardized diameter of 3–7 mm were placed (Fig. 1A–C). The superficial and deep cortexes were separated due to inherent differences in their elasticity values within these compartments [18]. The separation line between the superficial and deep cortexes was drawn in the middle of the cortex. The mean values of STE were chosen for data analysis. To assess the interobserver reproducibility of the STE measurements, two sonographers independently evaluated 30 randomly selected donor kidneys.

### Kidney histologic assessment

Following the STE examination, a 16-G automatic biopsy needle (Bard, Tempe, Arizona) was used to puncture the inferior pole of the kidney. All biopsy specimens were independently evaluated by two pathologists, each with over five years of experience. During the review process, they were blinded to the STE value. The chronic pathology of the biopsy tissues was graded according to the Remuzzi scoring system [4, 19], which considers features such as glomerulosclerosis (GS), interstitial fibrosis (IF), tubular atrophy (TA), and arteriosclerosis (AS). The Remuzzi score stratifies the biopsy into three groups (Fig. 1D–F): low (0–3), moderate (4–6), and high (7–12). Currently, acute tubular injury (ATI) is scored based on the Banff classification [20]. The grading of ATI is categorized as follows: mild, which includes epithelial flattening, tubule dilation, nuclear dropout, and loss of brush border; moderate, characterized by focal coagulative type necrosis; and severe, indicating infarction.

### KDPI

The kidney donor risk index (KDRI) is a method developed to measure the quality of kidney allografts [6]. KDPI is calculated by first determining the KDRI using various donor characteristics, including age, race, height, weight, stroke as the cause of death, donation after cardiovascular determination of death status, terminal serum creatinine (SCr), hepatitis C serostatus, and history of hypertension and diabetes.

### Follow-up and outcome measurement

All recipients were followed up for 6 months after transplantation. During the follow-up period, conventional medical treatments were administered in accordance with standard clinical practice, and allograft function became stabilized. The estimated glomerular filtration rate (eGFR) was calculated using the Cockroft–Gault formula, which incorporates SCr, age, gender, and body weight [21, 22].

### Statistical analysis

Normally distributed continuous variables were expressed as means ± standard deviations, while non-normally distributed continuous variables were presented as medians and interquartile ranges (IQRs). The comparison of STE values was performed using ANOVA or Pearson χ^2^ test. Correlations between variables were assessed using the Spearman correlation coefficient. Factor analysis was employed for dimensionality reduction analysis of collinear data [23]. The discriminative ability of variables was assessed through receiver operating characteristic (ROC) analysis.

The random forest algorithm was used to determine important predictive variables. Variables with an area under the receiver operating characteristic curve (AUC) exceeding 0.700 were considered to have acceptable discriminative ability and were used to plot ROC curves. Cut-off values were decided by using the Youden index. Interobserver agreement was assessed by using the intraclass correlation coefficient (ICC). Agreement was classified as poor (ICC < 0.4), moderate (ICC = 0.40–0.75), or excellent (ICC > 0.75) [24]. All statistical tests were performed by using R (version 4.3) or SPSS (version 22).

## Results

### Study population

**Fig. 2:**
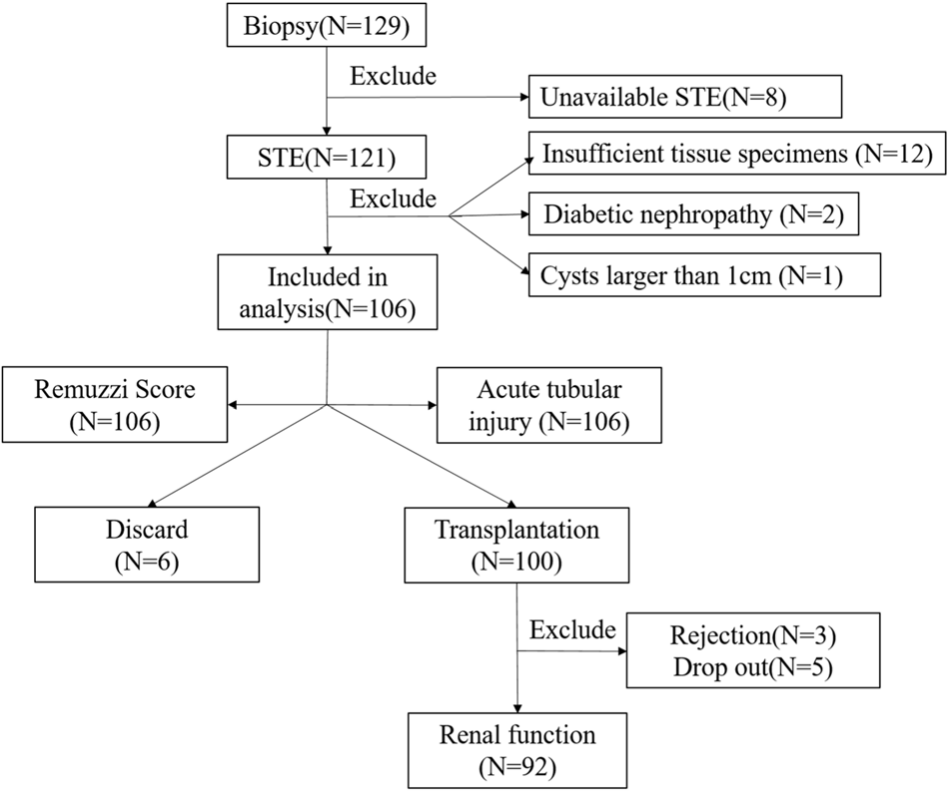
Flowchart of study design

From March 2022 to August 2023, a total of 129 donor kidneys that had undergone biopsy at our institution were prospectively enrolled. Among them, eight kidneys were not subjected to STE examination due to uncontrollable factors. Fifteen kidneys were excluded from the study, including twelve with insufficient specimens, two with diabetic nephropathy, and one with cysts larger than 1 cm in the middle pole. Ultimately, 106 donor kidneys were included in the study. The average age of donors was 46.2 ± 10.1 years, and males were dominant (*n* = 91, 85.8%). Following a comprehensive evaluation, six kidneys were discarded, primarily due to a high Remuzzi score. Consequently, a total of 100 donor kidneys were transplanted. To eliminate the influence of post-transplant factors on allograft function, five patients lost to follow-up and three patients experiencing acute rejection within the first six months were excluded from the study. Eventually, 92 recipients were included in the analysis for allograft function (Fig. 2). The enrolled recipients included 52 males and 40 females, with a mean age of 41.8 ± 12.1-years-old. Baseline characteristics are presented in Table 1.

**Table 1:** Baseline characteristics

Characteristic	Total	Low Remuzzi, (0–3)	Moderate Remuzzi, (4–6)	High Remuzzi, (7–12)	*p* value
Donor					
Age, (year)	46.2 ± 10.1	43.1 ± 10.6	50.3 ± 8.2^#^	45.9 ± 7.8^**,#^	0.002
Sex, (male/female)	91/15	44/11	36/2	11/2	0.133
BMI, (kg/m^2^)	24.5 ± 3.3	24.7 ± 4.4	24.9 ± 3.6	23.6 ± 2.2	0.466
Donor kidney, (DBD/DCD/living)	94/7/5	46/4/5	35/3/0	13/0/0	0.066
Diabetes, (yes/no)	15/91	2/53	10/28^**^	3/10^*^	0.005
Hypertension, (yes/no)	58/48	21/34	27/11^**^	10/3^*^	0.002
Scr, (μmol/L)	373.1 ± 193.5	388.6 ± 232.9	357.7 ± 138.0	391.9 ± 133.1	0.722
KDPI	64.8 ± 20.5	55.9 ± 21.2	74.4 ± 15.3^**^	74.5 ± 12.5^**^	< 0.001
Kidney length, (cm)	10.5 ± 0.8	10.5 ± 0.8	10.5 ± 0.9	10.4 ± 0.7	0.911
Kidney width, (cm)	5.1 ± 0.6	5.1 ± 0.6	5.0 ± 0.6	4.8 ± 0.7	0.189
Kidney					
IF (0/1/2/3)	40/54/10/2	40/15/0/0	0/36/2/0^**,#^	0/3/8/2^**,#^	< 0.001
TA (0/1/2/3)	41/58/5/2	41/14/0/0	0/37/1/0^**,#^	0/7/4/2^**,#^	< 0.001
GS (0/1/2/3)	29/52/19/6	26/25/4/0	3/26/9/0^**,#^	0/1/6/6^**,#^	< 0.001
AS (0/1/2/3)	41/45/9/11	38/17/0/0	3/26/4/5^**,#^	0/2/5/6^**,#^	< 0.001
ATI (1/2/3)	95/4/7	47/2/6	35/2/1	13/0/0	0.267
Parenchyma thickness, (cm)	1.93 ± 0.35	1.99 ± 0.23	1.97 ± 0.25	1.90 ± 0.24	0.500
Cold ischemia time, (h)	4.6 ± 1.5	4.5 ± 1.7	4.6 ± 1.3	5.1 ± 1.6	0.448
Warm ischemia time, (min)	0.7 ± 2.4	0.8 ± 2.5	0.7 ± 2.6	0.7 ± 2.8	0.553
Cortical echogenicity	38.6 ± 11.3	37.2 ± 10.8	39.6 ± 7.6^ǂ^	47.2 ± 10.4^**,ǂ^	0.005
STE_sc_, (kPa)	15.4 ± 5.4	13.2 ± 4.2	16.8 ± 2.9^**,#^	23.0 ± 4.6^**,#^	< 0.001
STE_dc_, (kPa)	24.9 ± 9.1	21.0 ± 5.5	26.8 ± 6.3^**,#^	39.5 ± 7.9^**,#^	< 0.001
STE_me_, (kPa)	39.3 ± 7.2	38.7 ± 3.8	40.0 ± 4.5^#^	46.6 ± 3.7^**,#^	< 0.001
Recipient					
Age, (year)	41.8 ± 12.1	40.5 ± 12.8	42.6 ± 10.8	42.4 ± 8.1	0.706
Gender, (male/female)	51/41	27/26	20/12	4/3	0.609
BMI, (kg/m^2^)	20.9 ± 3.6	20.4 ± 2.7	22.7 ± 4.3^**^	21.1 ± 3.9	0.015
Dialysis scheme, (no dialysis/hemodialysis/peritoneal dialysis)	5/64/23	3/38/12	2/21/9	0/5/2	0.857
Dialysis duration, (year)	2.3 ± 2.3	1.9 ± 2.3	2.8 ± 2.4	2.0 ± 1.6	0.260
DGF, (yes/no)	32/60	19/34	10/22	3/4	0.858
6-month, Scr	156.7 ± 74.3	135.9 ± 78.4	176.2 ± 49.5^*^	204.4 ± 114.3^*^	0.019

*BMI* body mass index, *DGF* delayed graft function, *DBD* donation after brain death, *DCD* donation after circulatory death, *KDPI* kidney donor profile index, *ATI* acute tubular injury, *STE* sound touch elastography

^*^ *p* < 0.05 vs low Remuzzi

^**^ *p* < 0.01 vs low Remuzzi

^ǂ^ *p* < 0.05 vs moderate Remuzzi or high Remuzzi

^#^ *p* < 0.01 vs moderate Remuzzi or high Remuzzi

### Interobserver agreement for ex vivo STE measurement

All ex vivo STE measurements were conducted successfully. There was excellent interobserver agreement in the STE measurements conducted by two sonographers, with ICC values of 0.843 (0.722–0.913), 0.904 (0.826–0.948), and 0.825 (0.692–0.903) for STE_sc_, STE_dc_, and STE_me_, respectively. The ICC value for cortical echogenicity (CE) was 0.923 (0.889–0.947) (Supplementary Table 1).

### Histopathologic and STE results

**Fig. 3:**
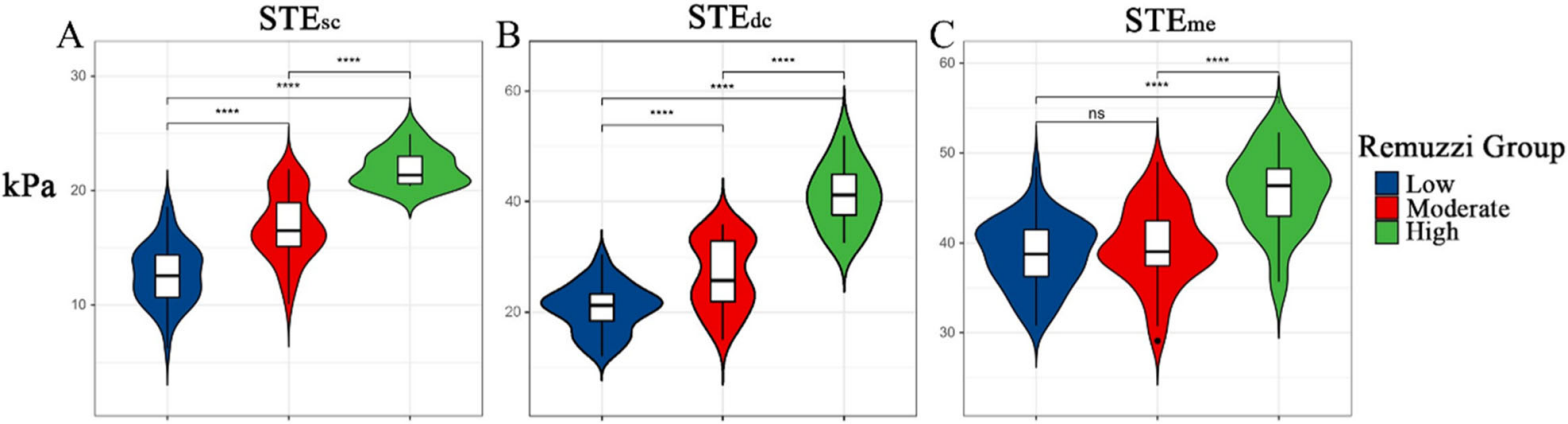
Violin plots showed the distributions of STE_sc_ (**A**), STE_dc_ (**B**), and STE_me_ (**C**) in the Remuzzi score. ***p<0.001

Regarding the Remuzzi score, the histopathological findings indicated that 55 kidneys were classified as low, 39 as moderate, and 12 as high. In the low, moderate, and high Remuzzi groups, the distribution of STE_sc_ was 13.2 ± 4.2, 16.8 ± 2.9, and 23.0 ± 4.6 kPa, respectively (*p* < 0.001) (Fig. 3A). Similarly, the distribution of STE_dc_ was 21.0 ± 5.5, 26.8 ± 6.3, and 39.5 ± 7.9 kPa, respectively (*p* < 0.001) (Fig. 3B). Likewise, the distribution of STE_me_ in the low, moderate, and high Remuzzi groups was 38.7 ± 3.8, 40.0 ± 4.5, and 46.6 ± 3.7 kPa, respectively (*p* < 0.001) (Fig. 3C). Moreover, the ATI examination revealed 95 cases with mild, 4 cases with moderate, and 7 cases with severe. Among the mild, moderate, and severe ATI groups, only STEme showed statistically significant differences, as determined by one-way ANOVA followed by post-hoc Tukey’s test (40.4 ± 4.4 kPa vs 35.8 ± 2.6 kPa vs 30.8 ± 4.8 kPa, *p* < 0.001) (Supplementary Fig. 2).

### Construction of STE using factor analysis

To explore the correlation between variables and Remuzzi or ATI, we conducted a Spearman correlation analysis. STE_sc_ (ρ = 0.666, *p* < 0.001), STE_dc_ (ρ = 0.587, *p* < 0.001), and STE_me_ (ρ = 0.374, *p* < 0.001) exhibited significant correlations with the Remuzzi score. More details regarding the correlation between STE and Remuzzi scores are listed in Table 2. No correlation was observed between STE_sc_ (ρ = −0.139, *p* = 0.157) and ATI, while STE_dc_ (ρ = −0.250, *p* = 0.01) and STE_me_ (ρ = −0.345, *p* < 0.001) were significantly correlated with ATI (Table 2). Furthermore, a correlation analysis was conducted for all variables, and the results are shown in Supplementary Fig. 3. We found a strong correlation among STE_sc_, STE_dc_, and STE_me_, with the KMO statistic being 0.608, which is greater than 0.5 (*p* < 0.001). To mitigate the impact of collinearity, we utilized the factor analysis method to extract key features and amalgamated these three variables into a single variable: STE = 0.152 × STE_sc_ + 0.160 × STE_dc_ + 0.138 × STE_me_. In the low, moderate, and high Remuzzi groups, STE exhibited distributions of 10.714 ± 1.770, 12.319 ± 1.823, and 16.244 ± 2.259, respectively. While in the mild, moderate, and severe ATI groups, the distributions of STE were 12.044 ± 2.395, 10.591 ± 2.285, and 9.928 ± 1.882, respectively.

**Table 2:** Correlation between donor characteristics and Remuzzi score or ATI

Characteristic	Remuzzi score		ATI	
	Spearman *r*	*p* value	Spearman *r*	*p* value
Gender	−0.200	0.040	−0.049	0.617
Age	0.332	0.001	−0.118	0.232
Kidney type	−0.280	0.004	−0.129	0.191
BMI	0.027	0.782	0.247	0.011
Diabetes	0.261	0.007	0.011	0.908
Hypertension	0.366	< 0.001	−0.038	0.703
Creatinine	0.005	0.963	0.164	0.095
Kidney length	0.073	0.479	0.021	0.841
Kidney width	−0.032	0.755	0.370	< 0.001
Cold ischemia time	0.178	0.068	−0.040	0.689
Warm ischemia time	−0.274	0.004	−0.129	0.191
Cortex thickness	−0.010	0.917	−0.004	0.969
Parenchyma thickness	−0.024	0.807	−0.062	0.532
Cortical echogenicity	0.376	< 0.001	−0.123	0.213
KDPI	0.408	< 0.001	−0.156	0.115
STE_sc_	0.666	< 0.001	−0.139	0.157
STE_dc_	0.587	< 0.001	−0.250	0.010
STE_me_	0.374	< 0.001	−0.345	< 0.001

*BMI* body mass index, *KDPI* kidney donor profile index, *STE* sound touch elastography, *ATI* acute tubular injury

### STE is predictive of the Remuzzi score

**Fig. 4:**
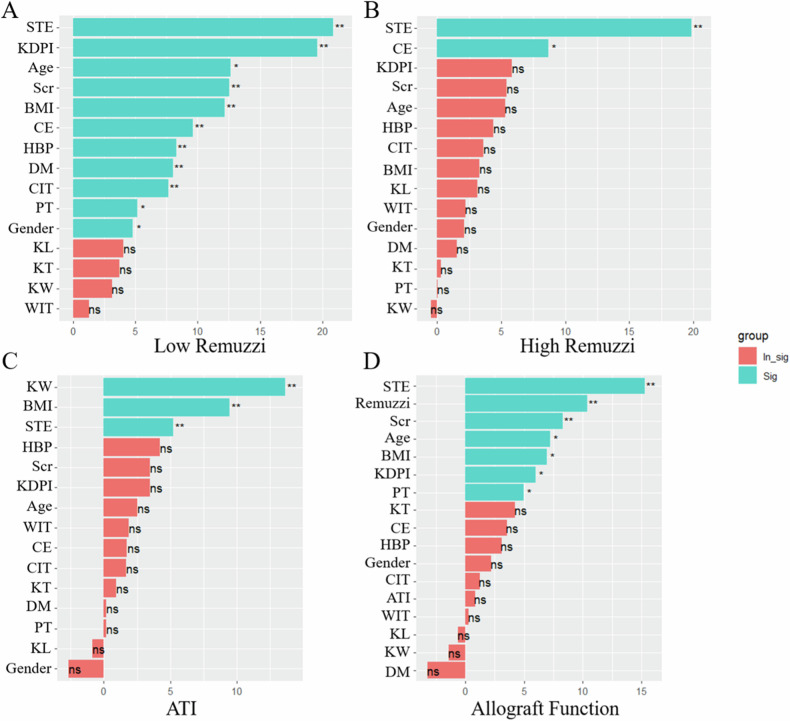
Importance ranking of characteristic variables. **A** Importance ranking of characteristic variables for low Remuzzi; **B** importance ranking of characteristic variables for high Remuzzi; **C** importance ranking of characteristic variables for ATI; and **D** importance ranking of characteristic variables for allograft function. **p < 0.01; *p < 0.05

According to the random forest algorithm, significant factors for the Remuzzi score and ATI are shown in Fig. 4A–C. Factors including STE, KDPI, age, SCr, BMI, CE, high blood pressure (HBP), DM, cold ischemia time (CIT), parenchyma thickness (PT), and gender were identified as key determinants for low Remuzzi score. Conversely, only STE and CE were determinant factors for a high Remuzzi score. As for ATI, kidney width (KW), BMI and STE were significant predictors.

**Fig. 5:**
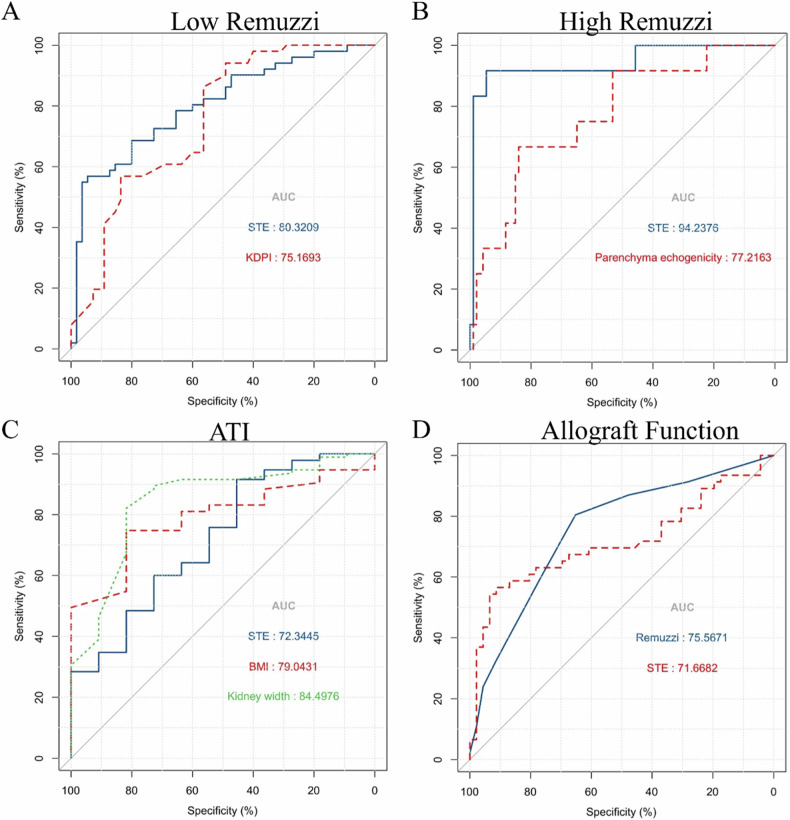
Predictive performance of STE. **A** Discrimination between low and moderate to high Remuzzi score; **B** discrimination between low to moderate and high Remuzzi score; **C** discrimination between low and moderate to high ATI; and **D** discrimination between good and poor allograft function

The diagnostic performance of each variable is listed in Table 3. Only factors with an AUC value exceeding 0.700 were included in the AUC curve analysis. The ROC curves depicting the variables for diagnosing the Remuzzi score and ATI are presented in Fig. 5A–C. The AUC for detecting low Remuzzi score based on STE was 0.803 (cutoff value, 48.74; sensitivity, 0.569; specificity, 0.945; PPV, 0.906; NPV, 0.703), while the AUC for KDPI was 0.752 (cutoff value, 54; sensitivity, 0.941; specificity, 0.491; PPV, 0.632; NPV, 0.900). For distinguishing high Remuzzi score, the AUC values were 0.772 for CE (cutoff value: 46.63; sensitivity: 0.667; specificity: 0.840; PPV: 0.348; and NPV: 0.952) and 0.943 for STE (cutoff value: 56.34; sensitivity: 0.917; specificity: 0.947; PPV: 0.688; and NPV: 0.989), with a statistically significant difference (*p* = 0.03). In differentiating mild ATI from moderate to severe ATI, the AUC for STE was 0.723 (cutoff value: 37.18; sensitivity: 0.916; specificity: 0.455; PPV: 0.935; and NPV: 0.385).

**Table 3:** Diagnostic performance for Remuzzi score and ATI

Characteristics	Threshold	AUC	Specificity	Sensitivity	PPV	NPV	*p* value
Low Remuzzi							
STE	12.589	0.803 (0.719–0.888)	0.945	0.569	0.906	0.703	–
KDPI	54	0.752 (0.659–0.844)	0.491	0.941	0.632	0.900	0.415
Age	40.5	0.675 (0.573–0.778)	0.491	0.882	0.616	0.818	0.046
BMI	27.718	0.507 (0.396–0.619)	0.941	0.218	0.800	0.527	< 0.001
CE	35.66	0.646 (0.539–0.752)	0.509	0.824	0.609	0.757	0.015
HBP	0.500	0.672 (0.582–0.761)	0.618	0.725	0.638	0.708	0.037
DM	0.500	0.609 (0.544–0.675)	0.964	0.255	0.867	0.582	< 0.001
CIT	3.250	0.552 (0.443–0.662)	0.236	0.922	0.528	0.765	< 0.001
Parenchyma thickness	1.875	0.553 (0.442–0.665)	0.510	0.673	0.597	0.591	< 0.001
Gender	0.500	0.561 (0.496–0.626)	0.922	0.200	0.733	0.516	< 0.001
High Remuzzi							
STE	14.567	0.943 (0.853–1.000)	0.947	0.917	0.688	0.989	–
CE	46.63	0.772 (0.629–0.916)	0.840	0.667	0.348	0.952	0.034
ATI							
STE	9.588	0.723 (0.558–0.889)	0.455	0.916	0.935	0.385	–
KW	5.35	0.845 (0.709–0.971)	0.821	0.818	0.346	0.975	0.230
BMI	25.463	0.790 (0.683–0.897)	0.747	0.818	0.273	0.973	0.381

*STE* sound touch elastography, *KDPI* kidney donor profile index, *BMI* body mass index, *HBP* high blood pressure, *DM* diabetes mellitus, *CIT* cold ischemia time, *CE* cortical echogenicity, *ATI* acute tubular injury, *PPV* positive predictive value, *NPV* negative predictive value, *KW* kidney width

*p* value: compared with STE on AUC

### STE is predictive of allograft function

During the 6-month follow-up period, 92 patients were included in the subsequent analysis of allograft function. According to the classification of eGFR, eGFR = 44 is used as the cutoff threshold [25]. The patients were referred into two groups: one group with good allograft function (eGFR ≥ 44) and the other group with poor allograft function (eGFR < 44). Random forest algorithm identified STE, Remuzzi score, SCr, Age, BMI, KDPI, and PT as important prognostic factors for 6-month eGFR (Fig. 4D). The prognostic performance of factors is summarized in Table 4. Only factors with an AUC value exceeding 0.700 were included in the subsequent analysis. Setting the threshold at 11.741, the AUC for STE was 0.717, with a sensitivity of 0.565, specificity of 0.913, PPV of 0.867, and NPV of 0.677. For Remuzzi score, when the threshold was set at 2.5, the AUC was 0.756, with a specificity of 0.652, sensitivity of 0.804, PPV of 0.698, and NPV of 0.769 (Fig. 5D). Although there was no significant difference in AUC (*p* = 0.518), the specificity of STE was significantly higher than that of Remuzzi score (*p* < 0.001).

**Table 4:** Predictive performance for 6-month allograft function

Characteristics	Threshold	AUC	Specificity	Sensitivity	PPV	NPV	*p* value	*p* value*
Remuzzi score	2.5	0.756 (0.657–0.855)	0.652	0.804	0.698	0.769	0.518	0.004
STE	11.741	0.717 (0.607–0.826)	0.913	0.565	0.867	0.677	–	–
KDPI	72.50	0.665 (0.553–0.777)	0.783	0.543	0.714	0.632	0.460	0.146
Age	37.50	0.649 (0.536–0.762)	0.326	0.957	0.587	0.882	0.315	< 0.001
BMI	25.30	0.614 (0.498–0.731)	0.804	0.457	0.700	0.597	0.181	< 0.001
Parenchyma thickness	2.00	0.565 (0.445–0.684)	0.696	0.522	0.632	0.593	0.082	< 0.001
SCr	176.0	0.531 (0.410–0.651)	0.196	1.000	0.554	1.000	0.019	< 0.001

*STE* sound touch elastography, *KDPI* kidney donor profile index, *BMI* body mass index, *SCr* serum creatinine (donor), *PPV* positive predictive value, *NPV* negative predictive value

*p* value: compared with STE on AUC

* *p* value: compared with STE on specificity

### Pre-transplant kidney quality evaluation based on STE

**Fig. 6:**
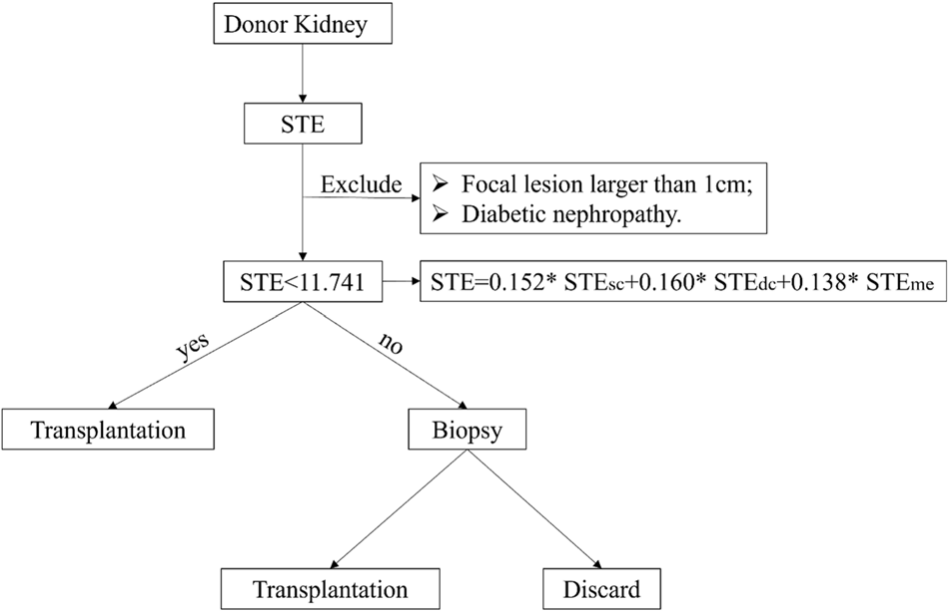
Donor kidney assessment scheme based on STE

To increase the utilization of marginal kidneys and reduce unnecessary biopsies, Fig. 6 illustrates our new method for evaluating pre-transplant kidney quality using ex vivo STE. When STE ≤ 11.741, the donor's kidney can be transplanted without the need for biopsy. Conversely, if STE exceeds 11.741, a reliable biopsy is unavoidable. Due to the high specificity of STE, we concluded that it can effectively select donor kidneys with a favorable prognosis after transplantation.

## Discussion

The Remuzzi score is the main reference for assessing the quality and prognosis of a kidney graft [4]. Currently, there is still a lack of non-invasive indicators that can effectively evaluate pre-transplant kidney quality. In our study, ex vivo STE emerged as a promising non-invasive indicator for assessing the Remuzzi score, with an AUC of 0.803 for diagnosing a low Remuzzi score and an AUC of 0.943 for diagnosing a high Remuzzi score. Importantly, ex vivo STE was employed to non-invasively predict the 6-month eGFR, yielding an AUC value of 0.717 and a specificity of 0.913. Based on these favorable results, we put forth a novel evaluation scheme for donor kidneys. When STE is ≤ 11.741, transplantation can be performed directly. When STE is greater than 11.741, a renal biopsy is required to determine whether the kidney should be transplanted or discarded. By utilizing this new scheme reasonably, we can significantly reduce the necessity for biopsies and minimize the wastage of marginal kidneys.

Currently, the utilization of elastography for ex vivo assessment of kidney quality is a completely novel approach. Previous studies primarily focused on conducting in vivo elasticity to assess donor kidney quality [26]. Although in vivo renal elasticity is more convenient, it is influenced by various confounding factors, such as BMI, anisotropy, measurement depth, transducer force, and so on [26, 27]. Moreover, several experimental studies have highlighted the significant impact of kidney perfusion on renal elasticity, with a contribution rate of up to 73% [15, 28]. In contrast, the ex vivo kidney was not perfused with blood and was unobstructed by subcutaneous tissue, allowing for flexible adjustment of elasticity angles. Therefore, in vitro measurement of renal elasticity can eliminate the interference of confounding factors and provide a more accurate reflection of the structural changes in donor kidneys. Furthermore, we gently placed the probe 2 mm above the kidney to prevent probe pressure so that no probe pressure was applied to the kidney. In addition, the absence of human factors such as breathing during ex vivo STE measurements contributed to excellent ICC in our study. Thus, in vitro measurement of renal elasticity had methodological feasibility and innovation.

STE had AUC values of 0.803 for diagnosing low Remuzzi and 0.943 for diagnosing high Remuzzi. Meanwhile, STE had an AUC of 0.723 for diagnosing moderate to severe ATI. This indicates that STE is particularly effective in reflecting the Remuzzi score, likely due to the significant weighting of STE_sc_ and STE_dc_ components within STE. For STE_sc_ and STE_dc_ performed well in the Remuzzi score classification. In contrast, STE_me_ demonstrated an advantage in identifying ATI, reflecting the renal medulla’s composition primarily consisting of renal tubules, which are profoundly affected by acute kidney injury [29].

Compared to the Remuzzi score, ex vivo STE measurement did not show superiority in predicting post-transplant renal function. However, it exhibited a significant advantage in terms of specificity by accurately identifying a “good” kidney. This advantage can be attributed to the avoidance of sampling errors that may occur during renal biopsy procedures. In our study, twelve cases were excluded from the analysis due to inadequate renal tissue sampling. Notably, the sampling area for elastography was significantly larger than that of renal biopsy. Additionally, we sampled three distinct regions based on the distribution of elastography images and employed factor analysis to extract primary features. This method enabled us to fully utilize the elastography information from each compartment and enhance the reliability of the STE measurement.

In the study, we focused on predicting the eGFR at 6 months post-transplant. This time point was chosen because allograft function tends to stabilize around the 6-month mark. Some kidney allografts may undergo DGF due to ischemia-reperfusion injury, which can take several months to recover [30]. Additionally, various factors, including recipient-related variables and postoperative complications, can influence post-transplant kidney function [31]. To assess the impact of donor-related factors on post-transplant allograft function, we excluded recipients who experienced complications, particularly episodes of acute rejection.

The ultimate objective of donor kidney assessment is to maximize donor utilization while minimizing unnecessary kidney biopsy [32]. Our proposed scheme for pre-transplant kidney quality assessment can effectively achieve this objective. Ex vivo STE measurements had high specificity for both the Remuzzi score and postoperative allograft function, indicating a strong capability to discern “good” kidneys.

Our study had several limitations. First, this was a single-center study with a relatively small sample size. Second, due to the absence of a validation group, the random forest algorithm was only used to select important variables rather than modeling. Finally, different elasticity techniques, such as Acoustic Radiation Force Impulse [33], may produce varying measurement results. Therefore, the application of alternative elasticity techniques in ex vivo kidney assessment would require revalidation.

## Conclusion

In conclusion, we suggested that the donor kidney can be transplanted directly when STE is less than 11.741. This can help avoid plenty of kidney biopsies, reducing the risk of bleeding complications. In cases where the STE exceeds 11.741, we recommended using biopsy for further evaluation to reduce the discard rate of donor kidneys. Ex vivo STE measurement shows promise in predicting postoperative kidney function and has the potential to reduce unnecessary renal biopsies in the future. Ex vivo STE may serve as a noninvasive predictor for assessing the quality of donor kidneys, which should be validated in further prospective studies.

## Supplementary information

ELECTRONIC SUPPLEMENTARY MATERIAL

## Data Availability

The datasets used for analyses during the current study are available from the corresponding author upon reasonable request.

## Abbreviations

ATI: Acute tubular injury
DGF: Delayed graft function
eGFR: Estimated glomerular filtration rate
ICC: Intraclass correlation coefficient
KDPI: Kidney donor profile index
SCr: Serum creatinine
STE: Sound touch elastography
