# Supplementary material for: Prediction of allograft function in pre-transplant kidneys using sound touch elastography (STE): an ex vivo study
Source: Insights Imaging. 2024 Oct 11;15:245. doi: 10.1186/s13244-024-01837-y (PMC11469982; doi:10.1186/s13244-024-01837-y)
Supplement: Supplementary file 1 — ELECTRONIC SUPPLEMENTARY MATERIAL [file 13244_2024_1837_MOESM1_ESM.pdf]

Prediction of Allograft Function in Pre-Transplant Kidneys Using Sound Touch Elastography (STE): An Ex Vivo Study

ELECTRONIC SUPPLEMENTARY MATERIAL

Supplementary Figure 1. Measurement of cortical echogenicity.

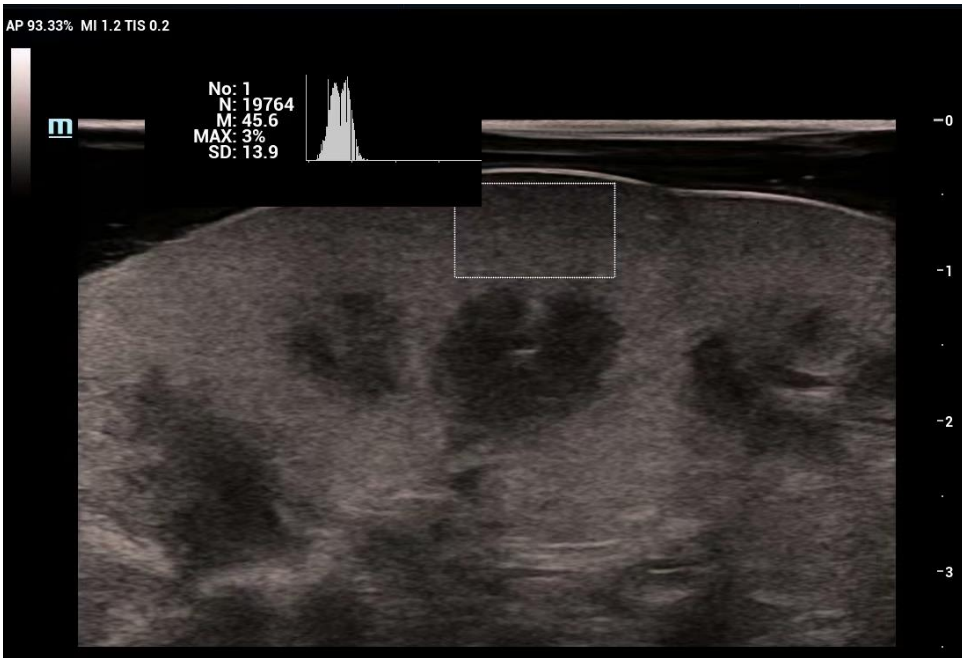

Supplementary Figure 2. Violin plots showed the distributions of STE_{sc}(A), STE_{dc}(B) and STE_{me}(C) in ATI.

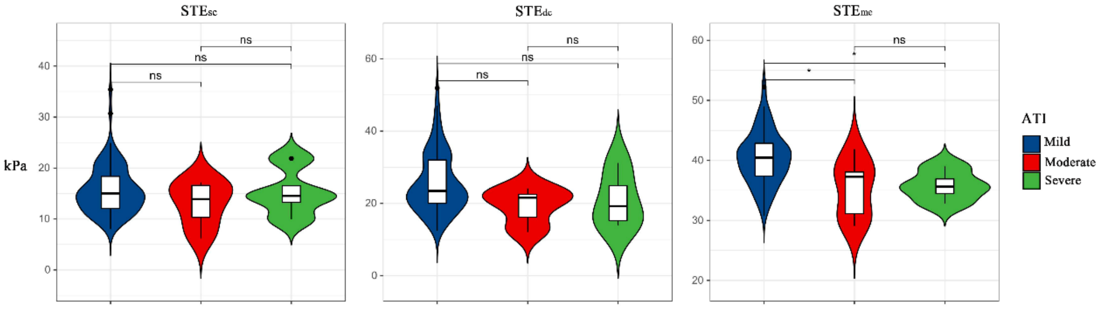

* p <0.05

Supplementary Figure 3. Correlation analysis for all variables.

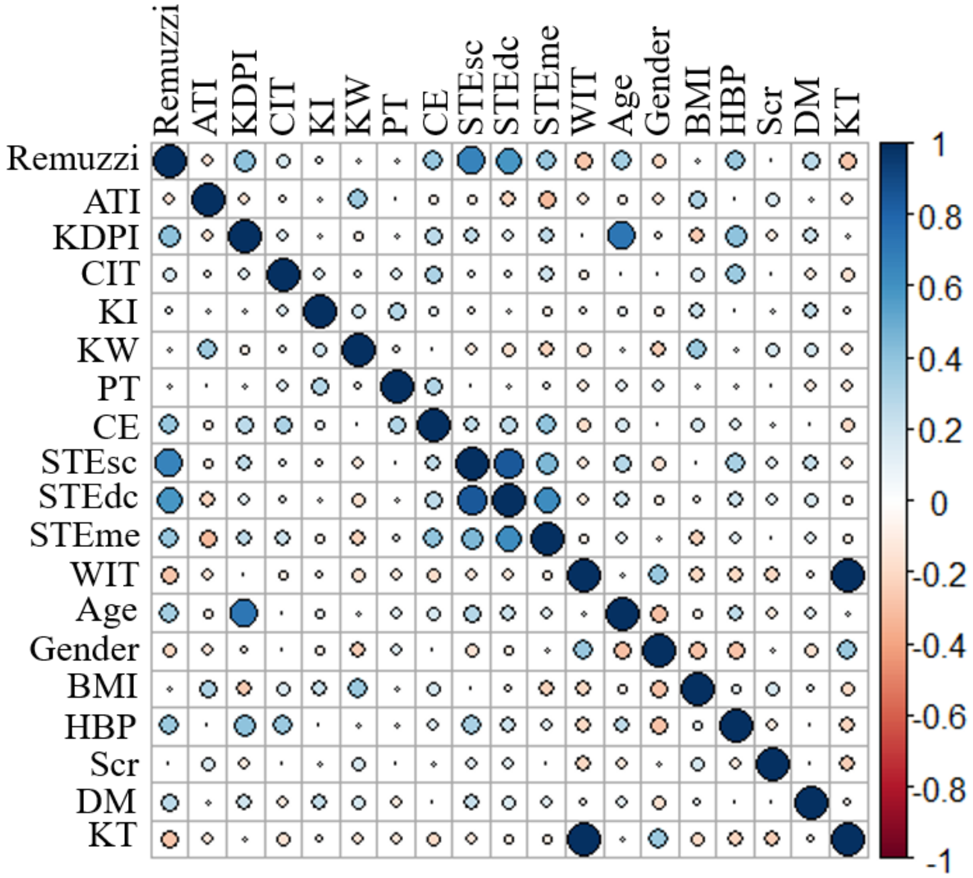

Supplementary table 1. The agreement of multimodality US features

Characteristic	ICC	*p* value	Agreement Level
STE_{sc}	0.843(0.722-0.913)	<0.001	Excellent
STE_{dc}	0.904(0.826-0.948)	<0.001	Excellent
STE_{me}	0.825(0.692-0.903)	<0.001	Excellent
Cortical echogenicity	0.923(0.889-0.947)	<0.001	Excellent

STE: Sound Touch Elastography; ICC: Intraclass Correlation Coeficient

Poor (ICC < 0.4), Moderate (ICC = 0.40–0.75), or Excellent (ICC > 0.75)
